# Predictive value of D4Z4 methylation levels for phenotypic heterogeneity and disease progression in Facioscapulohumeral Muscular Dystrophy with borderline D4Z4 repeat units: a retrospective cohort study

**DOI:** 10.7717/peerj.21043

**Published:** 2026-04-02

**Authors:** Xiaodan Lin, Qifang He, Minghui Zeng, Yuhua Lin, Xinrong Xu, Xinyue Chen, Xin Lin, Zhiqiang Wang

**Affiliations:** 1 Department of Neurology and Institute of Neurology of the First Affiliated Hospital, Institute of Neuroscience, and Fujian Key Laboratory of Molecular Neurology, Fujian Medical University, Fuzhou, Fujian, China; 2 Department of Neurology, Affiliated Mindong Hospital, Fujian Medical University, Fuan, Fujian, China; 3 Department of Neurology, National Regional Medical Center, Binhai Campus of the First Affiliated Hospital, Fujian Medical University, Fuzhou, Fujian, China

**Keywords:** Facioscapulohumeral muscular dystrophy, Borderline D4Z4 repeat units, DNA methylation, Phenotypic heterogeneity, Disease progression, SMCHD1 mutation

## Abstract

**Background:**

Facioscapulohumeral muscular dystrophy (FSHD) patients carrying borderline D4Z4 repeat units (DRUs) (8–10) represent a molecularly ambiguous group overlapping FSHD1 and FSHD2, characterized by pronounced phenotypic heterogeneity. This study aimed to determine the association between methylation levels and disease severity, progression in this undercharacterized cohort.

**Methods:**

This single-center and retrospective cohort study (ClinicalTrials.gov: NCT04369209), was conducted at the Fujian Neuromedical Centre (FNMC), China. Methylation levels were quantified using bisulfite sequencing in all participants. Whole-exome sequencing (WES) was performed for all probands. Phenotypic classification followed the Comprehensive Clinical Evaluation Form (CCEF). Motor function was assessed using the FSHD clinical score, clinical severity scale (CSS), and age-corrected CSS. Key functional endpoints were defined as follows: (1) lower extremity involvement, CSS ≥ 3.0; and (2) independent ambulation loss, CSS 4.5–5.

**Results:**

The patients carrying borderline DRUs exhibited milder phenotypes, broader phenotypic variability and lower methylation levels compared to those carrying 4–7 DRUs. The mean methylation levels of the 10 CpG sites and CpG6 methylation levels showed significant negative correlations with FSHD clinical score, CSS, and age-corrected CSS. Those methylation thresholds effectively discriminated symptomatic from asymptomatic patients with borderline DRUs. Furthermore, patients with lower methylation levels exhibited higher disease penetrance and an increased risk of progressing to lower extremity involvement. In a multigenerational pedigree, cooccurrence of a pathogenic *SMCHD1* variant exacerbated hypomethylation and clinical severity.

**Conclusions:**

Hypomethylation of the distal D4Z4 array serves as a robust biomarker for phenotypic penetrance and disease progression in borderline-allele FSHD. The co-presence of mutations in epigenetic modulators (*e.g.*, *SMCHD1*) and D4Z4 hypomethylation is correlated with more severe clinical phenotypes, underscoring a compound epigenetic-genetic disease mechanism.

## Introduction

Facioscapulohumeral muscular dystrophy (FSHD; OMIM 158900) is one of the most common neuromuscular dystrophies in adults, by an autosomal dominant inheritance pattern (Himeda & Jones, 2019; Ruggiero et al., 2020). Clinically, FSHD manifests as progressive weakness and atrophy initially affecting the facial, scapulohumeral, and humeral muscles, with subsequent involvement of pelvic girdle and axial musculature culminating, ultimately leading to loss of ambulation and wheelchair dependence in advanced stages (Katz et al., 2021; Qiu et al., 2022).

Genetically, FSHD classifies into two molecularly distinct yet mechanistically convergent subtypes: FSHD1, representing >95% of cases, arises from contraction D4Z4 repeat units (DRUs) (≤10) at the subtelomeric region of chromosome 4q35 in combination with the pathogenic 4qA haplotype (Wijmenga et al., 1992). FSHD2 is primarily associated with mutations in epigenetic regulators, such as *SMCHD1*, *DNMT3B*, and *LRIF1*, while the DRUs generally remain within the normal size range (≥11) (Lemmers et al., 2012, p. 2; Van den Boogaard et al., 2016; Hamanaka et al., 2020; Qiu et al., 2021, p. 2). Emerging perspectives suggest that FSHD1 and FSHD2 may represent a spectrum within a unified disease continuum (Sacconi et al., 2019). Notably, patients harboring borderline (DRUs) (8–10) occupy an interface between these two subtypes, exhibiting significant clinical heterogeneity. This subgroup is characterized by profound intrafamilial variability in phenotypic expression despite consistent disease-permissive DRUs (Ricci et al., 2020). Increasingly, this phenotypic heterogeneity is believed to be influenced by polygenic modifiers, complicating both prognostic prediction and genetic counseling.

Muscle biopsy studies have demonstrated that histopathological grading may serve as a useful quantitative marker of disease activity and severity in FSHD (Statland et al., 2015b). However, the invasive nature of muscle biopsies limits their utility for repeated clinical assessments and large-scale screening. At the molecular level, growing evidence suggests that DNA methylation levels at the distal D4Z4 repeat are inversely correlated with clinical severity (Zheng et al., 2023). Moreover, DNA methylation analysis has been incorporated into diagnostic testing by Clinical Laboratory Improvement Amendments (CLIA)-certified laboratories in the United States (Rieken et al., 2021), serving as a complementary biomarker to D4Z4 repeat units. While previous methylation studies have primarily focused on classical FSHD1 or FSHD2 cases, there has been limited investigation of the borderline DRUs, which presents substantial clinical heterogeneity. This study aims to fill this gap by focusing specifically on borderline alleles and their association with disease progression, providing a novel perspective on the role of D4Z4 methylation in this context.

In the present study, we conducted a retrospective cohort analysis to comprehensively characterize Chinese FSHD patients with borderline DRUs. By integrating clinical assessment and epigenetic genetic analyses, we aimed to advance precision diagnosis, enable more accurate prognostic stratification, and refine genetic counseling strategies for this distinctive patient population.

## Patients and Methods

### Study design and participants

**Figure 1:**
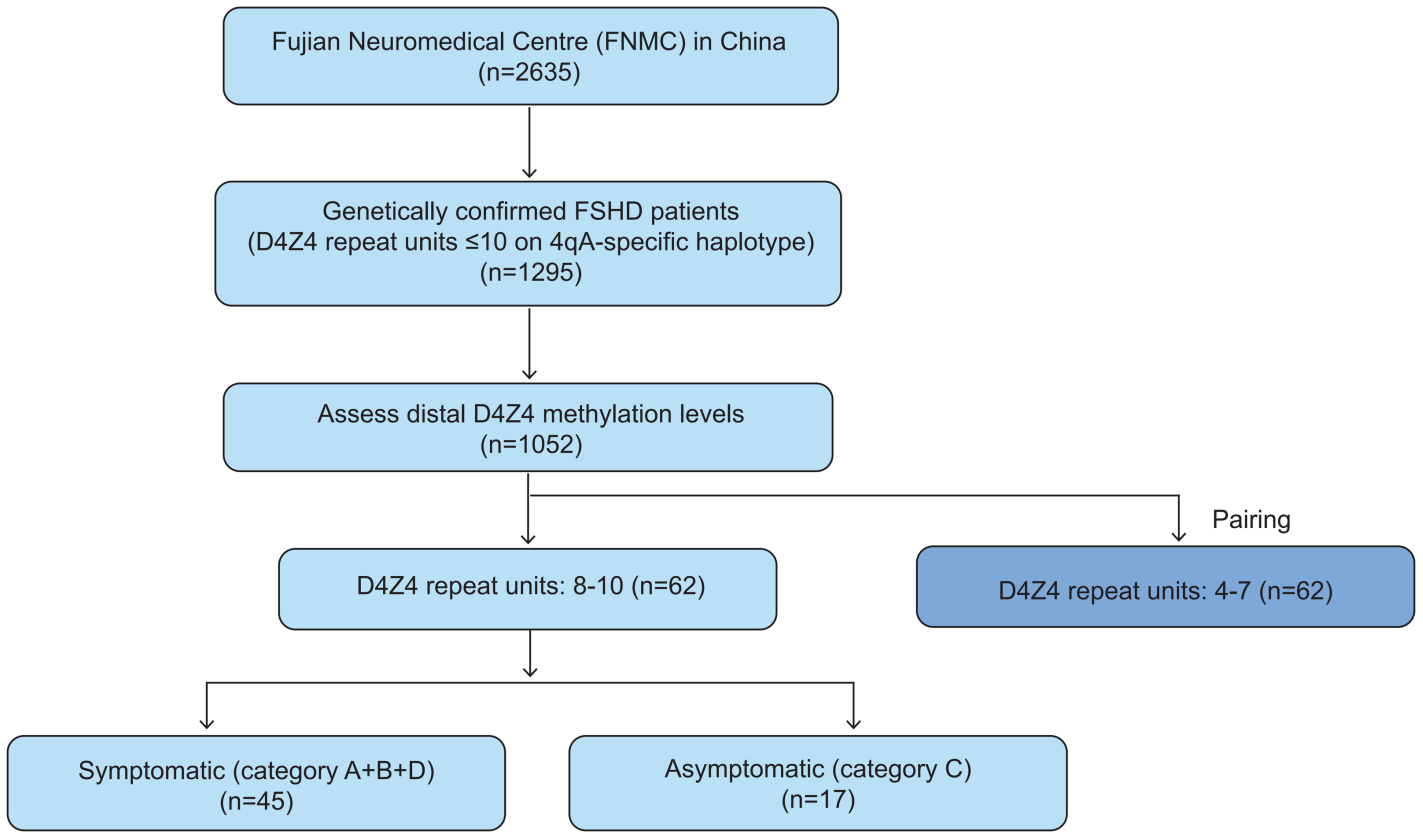
Flow diagram of patient recruitment and inclusion. Abbreviation: FSHD, facioscapulohumeral dystrophy; FNMC, Fujian Neuromedical Centre.

This was a single-center and retrospective cohort study, all participants were recruited from the FSHD registry (ClinicalTrials.gov, NCT04369209) at the Fujian Neuromedical Center (FNMC), China. Eligible participants were those with genetically confirmed FSHD, carried 1–10 DRUs on a 4qA-specific haplotype, with complete methylation sequencing data, recruited between January 2004 and January 2024. Participants were followed at clinically determined, variable intervals *via* outpatient visits or remote video consultations by the same neurologist (Z.Q.W.), ensuring consistency in clinical evaluation. Follow-up <1 year were excluded from longitudinal analyses. Participants with incomplete clinical datasets were excluded (Fig. 1).

### Standard protocol approvals and patient consent

The study protocol was approved by the Medical Research Ethics Committee of the First Affiliated Hospital, Fujian Medical University ([2023]403). Written informed consent was obtained from all participants in accordance with the principles outlined in the Declaration of Helsinki. The study was conducted in accordance with the Strengthening the Reporting of Observational Studies in Epidemiology (STROBE) guidelines.

### Clinical assessment

Baseline clinical data collected included sex, age at onset, age at first examination, and disease duration. Age at onset was defined as the time when the first-ever muscle weakness (Lamperti et al., 2010; Zheng et al., 2024). Clinical phenotypes were classified according to the 2016 Comprehensive Clinical Evaluation Form (CCEF) into four categories: patients with typical FSHD presenting facial and scapular girdle muscle weakness in category A; patients with muscle weakness limited to facial or scapular girdle muscles in category B; (3) asymptomatic subjects without motor impairment in category C; (4) patients with myopathic phenotype presenting other anomalous clinical features not consistent with FSHD in category D (Ricci et al., 2016). Motor function was assessed using the FSHD Clinical Score (Lamperti et al., 2010), the clinical severity scale (CSS), and the age-corrected CSS (Ricci et al., 1999; Overveld et al., 2005). Key functional endpoints were defined as follows: (1) lower extremity involvement, CSS ≥ 3.0; and (2) independent ambulation loss, CSS 4.5–5 (Zheng et al., 2023).

### Molecular genetic analysis

All participants underwent pulsed-field gel electrophoresis (PFGE) combined with Southern blotting, which confirmed a contraction of the DRUs on the pathogenic 4qA-specific haplotype (Calandra et al., 2016). DNA methylation level in the most distal D4Z4 region was quantified using bisulfite sequencing as previously described (Zheng et al., 2023). Whole-exome sequencing (WES) was performed for all probands to identify potential pathogenic variants. The resulting variants were prioritized based on silico variant predictors, minor allele frequency (MAF), the type of variant (coding, noncoding), and amino acid changes (if applicable). Suspected pathogenic variants segregating within the families were subsequently validated by Sanger sequencing. Finally, the variants were classified according to the American College of Medical Genetics (ACMG) guidelines and only variants likely pathogenic and pathogenic were reported in our study (Richards et al., 2015).

### Statistical analysis

Statistical analyses were conducted using SPSS software, version 25.0 (IBM Corp., Armonk, NY, USA) and GraphPad Prism (GraphPad Software, Inc., San Diego, CA, USA). Continuous variables not following a normal distribution were summarized as the median and range, whereas categorical variables were expressed as frequencies and percentages. Between-group comparisons were performed using the Mann–Whitney *U* test for continuous variables and the chi-square test for categorical variables; Fisher’s exact test was applied when the expected frequency in any cell was <5. Correlation analyses were carried out using Spearman’s rank correlation coefficient. Survival outcomes were evaluated by the Kaplan–Meier method, and differences between groups were assessed using the log-rank test. The area under the curve (AUC) of receiver operating characteristic (ROC) curves was used to evaluate the discriminatory ability of distal D4Z4 hypomethylation within the 4qA-specific allele between symptomatic and asymptomatic FSHD patients. All statistical tests were two-tailed, and a *p*-value <0.05 was considered indicative of statistical significance.

## Results

### Baseline characteristics of patients with 8–10 DRUs

A total of 62 FSHD patients carrying 8–10 DRUs from 39 unrelated families were included in the final analysis. A control group carrying 4–7 DRUs was established by matched based on sex, age, and disease duration (Table 1). Among patients with 8–10 DRUs, 33 (53.2%) were male. The median age at symptom onset was 22 years (range, 12–67 years), and the median age at first examination was 38 years (range, 12–76 years). According to the CCEF classification, 34 patients (54.8%) were classified as category A, 10 (16.1%) as category B, 17 (27.4%) as category C, and 1 (1.6%) as category D. The median mean methylation levels of the 10 CpG sites was 44.19% (range, 25.91–80.24%) and the median CpG6 methylation levels was 68.33% (range, 34.40–96.84%). The median FSHD clinical score was 4 (range, 0–12), the median CSS was 1.5 (range, 0–4), and the median age-corrected CSS was 107.14 (range, 0–400). Lower extremity involvement was observed in 27 patients (43.5%), with a median age at onset of 36 years (range, 15–68 years) and a median disease duration of 7 years (range, 0–50 years).

**Table 1:** Baseline characteristics between 8–10 DRUs and 4–7 DRUs group.

	**8–10 DRUs group**	**4–7 DRUs group**	***p*** ** value**
**Demographics**			
Participants, n	62	62	
Sex, male, n (%)	33 (53.2)	33 (53.2)	1^a^
Age at symptom onset, y, median (range)	22 (12–67)	20 (7–46)	0.112^b^
Age at first examination, y, median (range)	38 (12–76)	37 (9–68)	0.877^b^
Disease duration from onset, y, median (range)	8 (0–47)	17 (2–39)	0.140^b^
**Clinical characteristics**			
Phenotypic classification (CCEF), n (%)			
Category A	34 (54.8)	50 (80.6)	**0.01** ^a^
Category B	10 (16.1)	5 (8.1)	
Category C	17 (27.4)	6 (9.7)	
Category D	1 (1.6)	1 (1.6)	
**Genetic characteristics**			
Distal D4Z4 methylation level, median (range %)			
CpG1 locus	23.99 (3.86–60.60)	23.54 (9.81–49.19)	0.276^b^
CpG2 locus	59.86 (32.69–94.42)	54.64 (31.51–78.99)	0.074^b^
CpG3 locus	20.16 (9.59–57.44)	16.68 (9.58–32.31)	**0.011** ^b^
CpG4 locus	41.91 (20.1–82.45)	37.56 (14.41–59.24)	**0.047** ^b^
CpG5 locus	36.93 (14.42–77.64)	29.96 (12.7–50.79)	**0.003** ^b^
CpG6 locus	68.33 (34.40–96.84)	60.28 (25.08–87.53)	**0.004** ^b^
CpG7 locus	58.99 (33.91–96.73)	53.24 (27.63–72.12)	**0.015** ^b^
CpG8 locus	49.19 (8.82–7.60)	44.20 (24.33–70.22)	0.087^b^
CpG9 locus	40.22 (293–74.34)	35.75 (1.76–65.08)	**0.028** ^b^
CpG10 locus	42.20 (22.04–83.56)	39.19 (15.60–65.44)	0.088^b^
Average methylation level of the 10 CpG sites	44.19 (25.91–80.24)	38.92 (19.72–56.66)	**0.015** ^b^
**Motor function**			
FSHD clinical score (0–15), median (range)	4 (0–12)	7 (0–12)	**<0.001** ^b^
CSS (0–5), median (range)	1.5 (0–4.0)	3 (0–4.5)	**0.005** ^b^
Age-corrected CSS (0–10000), median (range)	107.14 (0–400)	144. 60 (0–380.95)	**0.004** ^b^
Lower extremity involvement, n (%)	27 (43.5)	42 (67.7)	**0.007** ^a^
Onset age of lower extremity involvement, y, median (range)	36 (15–68)	32 (16–6)	0.13^b^
Duration from onset to lower extremity involvement, y, median (range)	7 (0–50)	7 (0–29)	0.587^b^

**Notes.**

a *p* values were based on *χ*^2^ test (or Fisher’s exact test) for frequencies.

b *p* value were based on Mann–Whitney *U* test for continuous variables.

Values in bold indicate *p* value <0.05.

Abbreviations FSHD Facioscapulohumeral muscular dystrophy type CCEF Comprehensive Clinical Evaluation Form CSS Clinical severity scale

### Assessment of disease severity between the 8–10 DRUs and 4–7 DRUs groups

Compared with patients carrying 4–7 DRUs, those with 8–10 DRUs presented with significantly milder clinical phenotypes. Specifically, they had lower median FSHD clinical scores (4 *vs.* 7, *p* < 0.001), lower median CSS (1.5 *vs.* 3.0, *p* = 0.005), and lower median age-corrected CSS (107.14 *vs.* 144.60, *p* = 0.004). Patients with 8–10 DRUs also exhibited a broader phenotypic spectrum according to the CCEF classification (*p* = 0.01). Furthermore, the proportion of patients with lower extremity involvement was significantly lower in the 8–10 DRUs group compared with the 4–7 DRUs group (43.5% *vs.* 67.7%, *p* = 0.007).

### Correlation between methylation levels and disease severity and progression

**Figure 2:**
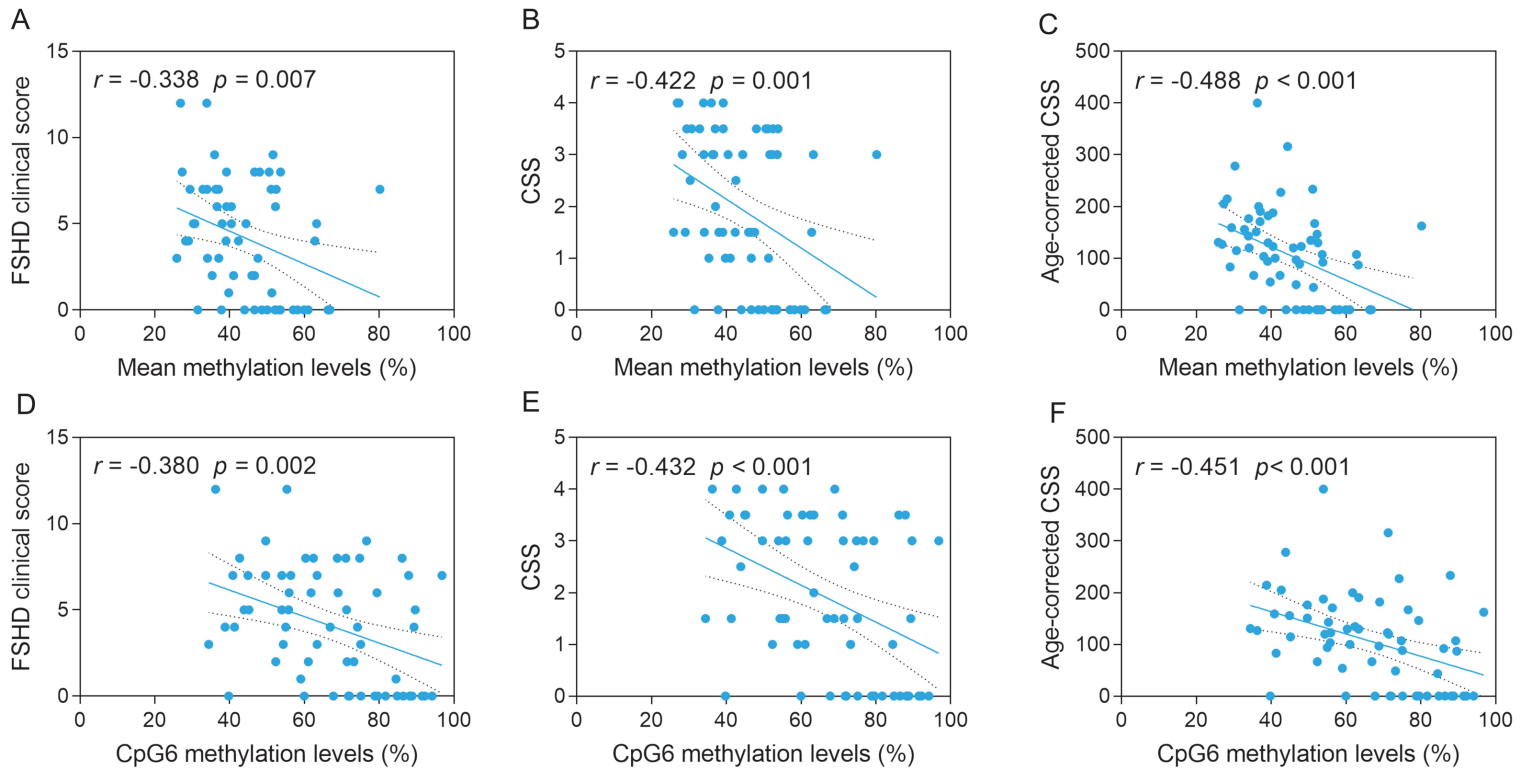
Associations for disease severity scores with methylation levels for the borderline DRUs FSHD patients. (A, B, C) Correlations (Spearman’s rank) between FSHD clinical score (A, *r* =  − 0.338, *p* = 0.007), CSS (B, *r* =  − 0.422, *p* = 0.001) and age-corrected CSS (C, *r* =  − 0.488, *p* < 0.001) and the mean methylation levels of the 10 CpG sites for the borderline DRUs FSHD patients. (D, E, F) Correlations (Spearman’s rank) between FSHD clinical score (A, *r* =  − 0.380, *p* = 0.002), CSS (B, *r* =  − 0.432, *p* < 0.001) and age-corrected CSS (C, *r* =  − 0.451, *p* < 0.001) and CpG6 methylation levels for the borderline DRUs FSHD patients.

**Figure 3:**
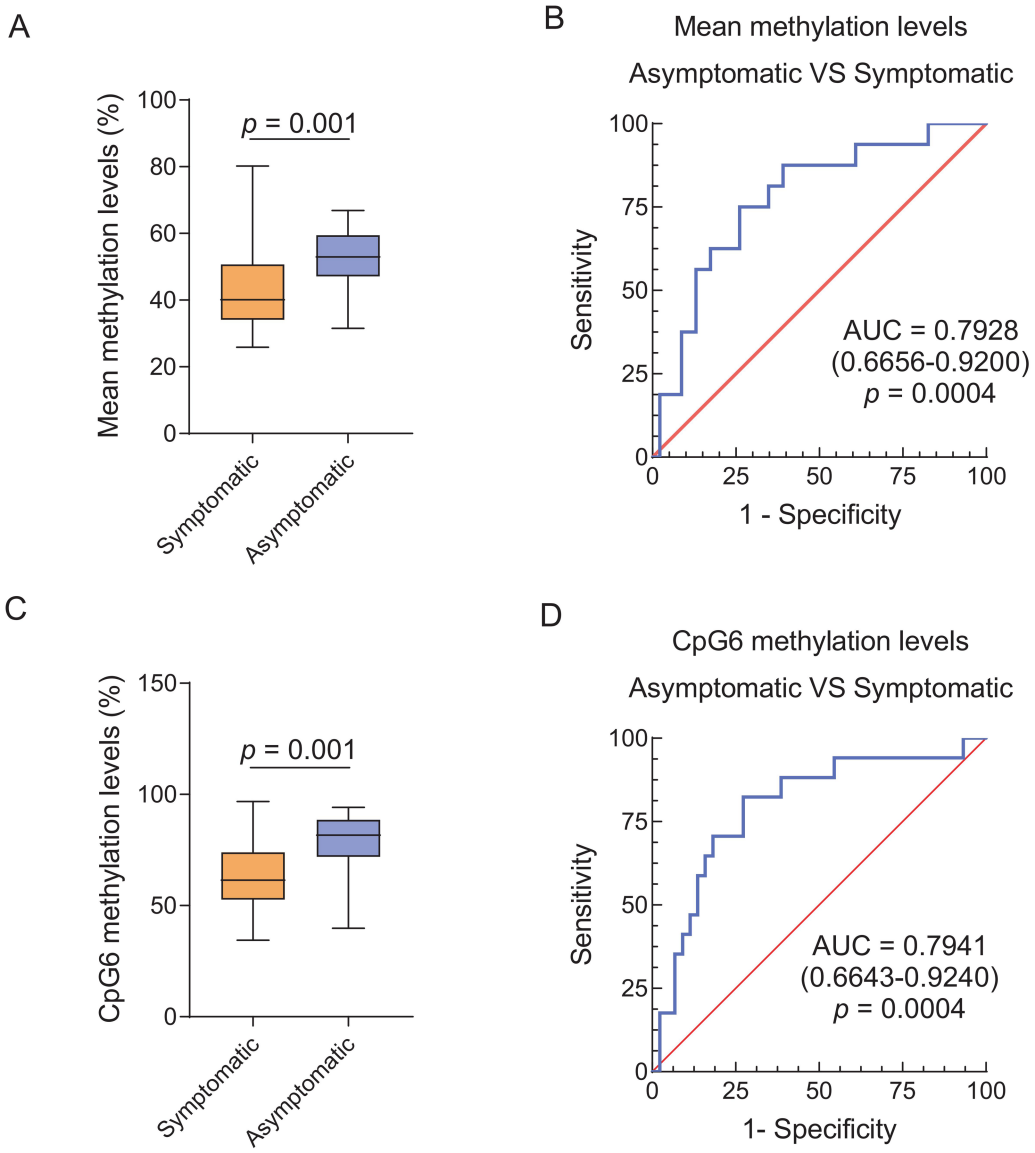
Differences in methylation levels between symptomatic and asymptomatic carriers with borderline DRUs. (A, C) Significant differences in the mean methylation levels of the 10 CpG sites (A, *p* = 0.001) and CpG6 methylation levels (C, *p* = 0.001) between symptomatic FSHD patients and asymptomatic carriers with borderline DRUs. (B, D) ROC curve analyses for differentiating between symptomatic FSHD patients and asymptomatic carriers with borderline DRUs according to the mean methylation levels of the 10 CpG sites (B, AUC of 0.7928 (95% CI [0.67–0.92]), *p* = 0.0004) and CpG6 methylation levels (D, AUC of 0.7941 (95% CI [0.66–0.92]), *p* = 0.0004). FSHD, facioscapulohumeral dystrophy type; ROC, receiver operating characteristic; AUC, area under the curve.

**Figure 4:**
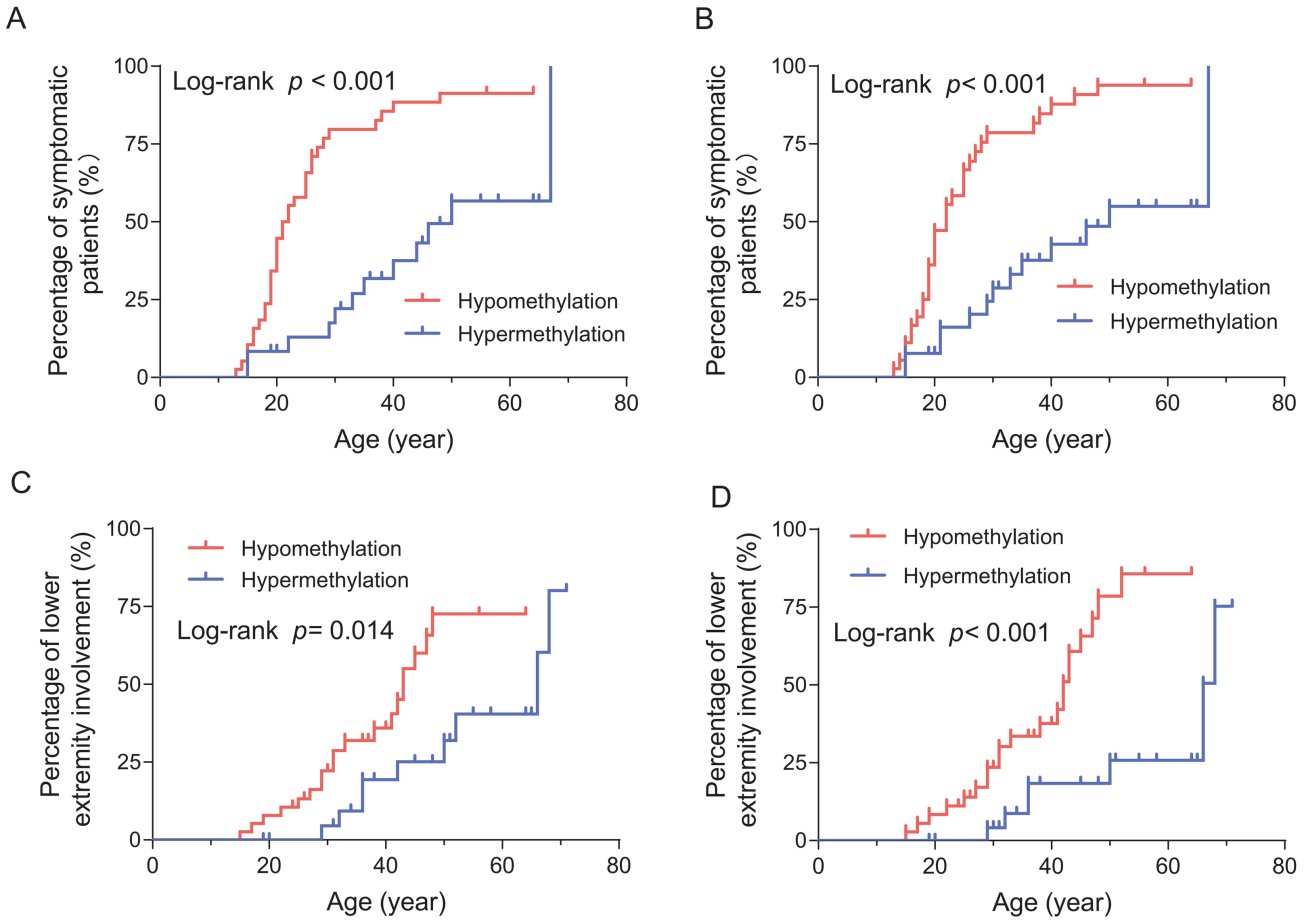
Kaplan–Meier curves for age at onset and lower extremity involvement in hypomethylation and hypermethylation patients with borderline DRUs. (A, B) Significant differences in age at onset and lower extremity involvement between hypomethylation and hypermethylation patients were analyzed with an optimal cutoff value of 48.9% of the mean methylation level of the 10 CpG sites by log-rank test (age at onset, *p* < 0.001; lower extremity involvement, *p* = 0.0 14). (C, D) Significant differences in age at onset and lower extremity involvement between hypomethylation and hypermethylation patients were analyzed with an optimal cutoff value of 71.6% of CpG6 methylation levels by log-rank test (both, *p* < 0.001).

The mean methylation levels of the 10 CpG sites were negatively correlated with the FSHD clinical score (*r* =  − 0.338, *p* = 0.007), CSS (*r* =  − 0.422, *p* = 0.001) and age-corrected CSS (*r* =  − 0.488, *p* < 0.001). Similarly, the CpG6 methylation levels demonstrated a negative correlation with the FSHD clinical score (*r* =  − 0.380, *p* = 0.002), CSS (*r* =  − 0.432, *p* < 0.001) and age-corrected CSS (*r* =  − 0.451, *p* < 0.001) (Fig. 2). Patients with 8–l0 DRUs were divided into symptomatic (categories A, B, and D) and asymptomatic (category C) subgroups based on the CCEF classification. Both the mean methylation levels of the 10 CpG sites and CpG6 methylation levels were significantly higher in asymptomatic subgroup compared with the symptomatic group (both *p* = 0.001) (Figs. 3A, 3C). ROC curve analysis revealed that the mean methylation level of the 10 CpG sites discriminated between symptomatic and asymptomatic patients with an AUC of 0.7928 (95% CI [0.67–0.92]) and an optimal cutoff value of 48.9%. Similarly, CpG6 methylation levels yielded an AUC of 0.7941 (95% CI [0.66–0.92]) and an optimal cutoff value of 71.6% (Figs. 3B, 3D). To further investigate the association between D4Z4 methylation status and age at onset and disease progression, patients with 8–10 DRUs were stratified into hypomethylation and hypermethylation subgroups based on these ROC-derived cutoff values. Kaplan–Meier survival analyses demonstrated that the hypomethylation subgroup had a significantly earlier age at onset (log-rank test, both *p* < 0.001) (Figs. 4A, 4B) and a markedly increased cumulative incidence of lower extremity involvement (log-rank test, the mean methylation level of the 10 CpG sites, *p* = 0.014; CpG6 methylation levels, *p* < 0.001) (Figs. 4C, 4D). After an average follow-up period of 5years, a total of 52 patients with 8–10 DRUs were included in the follow-up analysis. Among them, two patients in the hypomethylation subgroup progressed to lower extremity involvement, and two progressed to loss of independent ambulation, No patients in the hypermethylation subgroup reached either of these endpoints. Detailed follow-up data for both subgroups were summarized in Table S1.

### Analysis of suspected pathogenic variants

**Figure 5:**
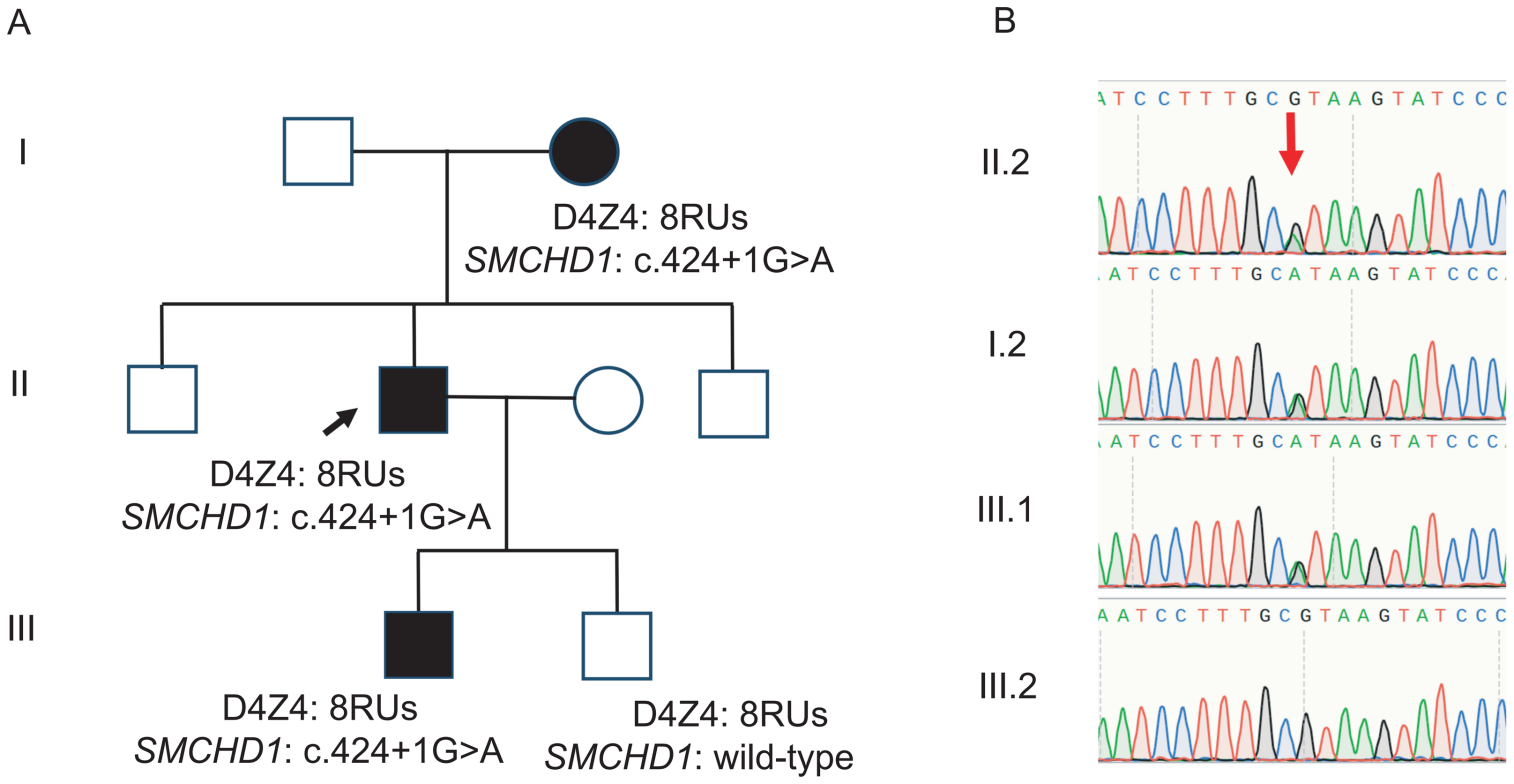
Pedigree and Sanger sequencing of the FSHD family. (A) Pedigree of the FSHD family, D4Z4 repeat arrays, *SMCHD1* variation, are shown. Note that the father (I.1), the brothers (II.1, II.3) declined to give blood samples. (B) Sanger sequencing verified the heterozygous nucleotide variant (c.424+1G>A) in *SMCHD1* in the FSHD family.

In this study, a pathogenic splice-site mutation in the *SMCHD1* (c.424+1G>A) was identified in an FSHD family carrying eight DRUs (Fig. 5) (Lemmers et al., 2015). The proband (II.2, a 44-year-old man), his mother (I.2, a 63-year-old woman), and his elder son (III.1, a 18-year-old man) carried the pathogenic *SMCHD1* mutation and exhibited classical FSHD-associated muscle weakness with FSHD clinical score of 7, 12, and 5, respectively. All three individuals displayed markedly reduced D4Z4 methylation levels, the mean methylation levels of the 10 CpG sites were 29%, 27%, and 30%; CpG6 methylation levels were 44%, 36%, and 41%, respectively (Table 2). In contrast, the younger son (III.2, a 12-year-old boy), who also carried eight DRUs but did not harbor the *SMCHD1* mutation, exhibited substantially higher methylation levels and remained clinically asymptomatic at the time of the study. After the 7-year follow-up, the proband, his mother, and his elder son demonstrated FSHD clinical scores of 10, 14, and 7, respectively.

## Discussion

Although FSHD was historically classified as a monogenic disorder resulting from D4Z4 repeat contraction, more recent studies established it as fundamentally an epigenetic disease with complex regulatory pathology (Sacconi et al., 2019; Rieken et al., 2021). The 268th ENMC International Workshop on FSHD diagnostic consensus emphasized that patients carrying 8–10 DRUs occupied a borderline position and a molecular overlap region between FSHD1 and FSHD2 (Montagnese et al., 2023; Giardina et al., 2024). Notably, a broad range of phenotypic variability was observed among patients with high numbers of DRUs, spanning from asymptomatic carriers to individuals with severe disability and wheelchair dependence. This variability represented a significant “blind spot” in clinical management.

In this study, we performed the first systematic analysis of the clinical and epigenetic characteristics of patients carrying borderline DRUs in a Chinese FSHD cohort. Consistent with previous reports, most individuals in this subgroup exhibited a later age at onset, milder symptoms, lower methylation levels (Statland et al., 2015a), and a higher proportion of asymptomatic carriers. Although some carriers of borderline DRUs presented with the typical facial and scapular girdle muscle weakness pattern (category A), a considerable proportion exhibited clinical manifestations that deviated from the classical phenotype (Ruggiero et al., 2020). Previous studies consistently reported a negative correlation between the number of DRUs and disease severity (Katz et al., 2021). However, the marked phenotypic heterogeneity observed in this subgroup indicated that D4Z4 repeat number alone was insufficient for diagnostic or prognostic stratification. Integration of a comprehensive genetic background analysis was therefore considered essential for accurate clinical evaluation and management.

**Table 2:** Suspected pathogenic variants and genetic signatures.

**Patient ID**	**Age at first examination, y**	**CCEF category**	**FSHD clinical score at baseline**	**FSHD clinical score at follow-up**	**D4Z4 repeat units**	**Mean methylation** **levels (%)**	**CpG6 methylation** **levels (%)**	**WES variants**	**ACMG classification**
I.2	63	A1	12	14	8	27	36	*SMCHD1*: c.424+1G >A	likely pathogenic
II.2	44	A2	7	10	8	29	44	*SMCHD1*: c.424+1G >A	likely pathogenic
III.1	18	A1	5	7	8	30	41	*SMCHD1*: c.424+1G >A	likely pathogenic
III.2	12	C2	0	0	8	67	94	wild-type	–

**Notes.**

Abbreviations FSHD Facioscapulohumeral muscular dystrophy type CCEF Comprehensive Clinical Evaluation Form WES Whole-exome sequencing ACMG American College of Medical Genetics

Specifically, we acknowledged that previous studies have shown strong associations between D4Z4 DNA methylation levels and disease severity and progression in FSHD (Rieken et al., 2021; Zheng et al., 2023). However, the role of DNA methylation in the relatively poorly understood borderline DRUs subgroup remained insufficiently explored. In the present study, we specifically focused on this subgroup and found that, among patients carrying borderline DRUs, asymptomatic carriers exhibited significantly higher methylation levels compared to symptomatic individuals. Moreover, methylation levels displayed a negative correlation with the FSHD clinical score, CSS, and age-corrected CSS. In this study, ROC and Kaplan–Meier analyses were explicitly conducted as exploratory approaches to further assess the potential utility of methylation levels as indicators of disease progression. The ROC curve analysis identified quantitative cut-off values, suggesting that lower methylation levels were associated with an earlier age at onset and an increased risk of progressing to lower extremity involvement. Within this specific cohort, these findings demonstrated that methylation levels might aid in discriminate between symptomatic and asymptomatic carriers. Consistent with previous reports (Rieken et al., 2021), our results suggested that methylation status provided a more informative predictor of disease progression in individuals with borderline DRUs, thereby challenging traditional diagnostic models based solely on repeat number. However, it was important to emphasize that the methylation thresholds were derived and evaluated within the same dataset, which introduced a potential risk of overfitting. Therefore, these cut-off values should not be interpreted as clinically actionable at this stage. Further longitudinal investigations and external validation in independent cohorts were required before methylation-based metrics could be considered for clinical application.

After a mean follow-up of approximately 5 years, patients in the hypomethylation subgroup showed a relatively greater tendency for clinical progression compared to those in the hypermethylation subgroup. This difference might primarily stem from the slow and heterogeneous progression of FSHD, especially within the 8–10 DRU range, which limited the ability to detect a noticeable divergence over a relatively short observation period (Dijkstra et al., 2021; Dijkstra et al., 2025). Future studies should therefore focus on patient subgroups with more rapid disease progression and incorporate longer-term follow-up to more accurately characterize methylation-associated differences in clinical trajectories (Goselink et al., 2019).

In addition, this study identified a pathogenic variant in the *SMCHD1* (c.424+1G>A) within a representative pedigree, and established a definite association between this mutation, hypomethylation, and severe clinical phenotypes (Lemmers et al., 2015). This finding was consistent with the previously proposed “digenic inheritance” model, in which D4Z4 repeat contraction characteristic of FSHD1 coexisted with mutations in FSHD2 modifier genes in the same individual, exerting synergistic pathogenic effects (Sacconi et al., 2013; Larsen et al., 2015). In this family, carriers of the same 8 DRUs who also harbored the *SMCHD1* mutation consistently exhibited lower methylation levels and more severe phenotypes, whereas relatives lacking the mutation maintained higher methylation levels and remained asymptomatic over extended follow-up periods. These findings underscored the critical role of epigenetic regulation in determining phenotypic penetrance and highlighted the potential value of comprehensive clinical evaluation that includes both D4Z4 methylation levels and pathogenic variants in epigenetic modifier genes, particularly in individuals exhibiting discordant genotype–phenotype correlations. However, it should be emphasized that the *SMCHD1* pedigree represented a single family, which substantially limited the generalizability of these observations. Accordingly, these results should be interpreted with caution. Future studies involving larger and more diverse cohorts would be necessary to validate the proposed digenic interaction and to further elucidate the underlying mechanisms across different populations.

This study has several limitations that warranted careful consideration. First, the single-center, retrospective design, combined with recruitment primarily from a clinically presenting patient population, might have introduced selection bias toward more severe phenotypes. This could lead to an overestimation of disease penetrance and an accelerated progression timeline in our time-to-event analyses. Second, the methylation cutoffs were derived and evaluated within the same dataset, increasing the risk of overfitting and limiting the external validity and generalizability of the identified thresholds. Third, although whole-exome sequencing was performed for all probands, the absence of family co-segregation analyses and functional validation limited the systematic evaluation of the pathogenicity and clinical significance of potential disease-causing variants. Consequently, the genotype-phenotype correlation analyses were constrained. Future research should focus on: (1) establish multicenter, prospective patient registry systems incorporating standardized clinical assessment scales for consistent phenotypic classification to improve the accuracy and generalizability of findings; (2) external validation using independent cohorts and, where possible, longitudinal designs to assess the reproducibility and robustness of these methylation thresholds across diverse populations. (3) expand recruitment to include complete pedigrees or multiple affected generations, which would facilitate co-segregation analyses to elucidate inheritance patterns and provide more robust evidence for genetic counseling and disease management.

## Conclusion

In summary, our study provided the first systematic characterization of clinical heterogeneity and epigenetic regulation in FSHD patients with borderline DRUs from a Chinese cohort, and offered integrated evidences supporting the disease continuum model. These findings contributed new insights advanced the development of a precision medicine framework for FSHD, and established a solid clinical foundation for individualized management and genetic counseling in patients carrying borderline DRUs.

##  Supplemental Information

10.7717/peerj.21043/supp-1 Supplemental Table 1 Comparison between hypomethylation and hypermethylation patients at baseline and follow-up based on CpG6 methylation levels

10.7717/peerj.21043/supp-2 Supplemental Information 2 Raw data

## Additional Information and Declarations

### DNA Deposition

The following information was supplied regarding the deposition of DNA sequences: The single nucleotide variant is available at ClinVar: VCV000497570.7.

### Data Availability

The following information was supplied regarding data availability: The raw data is available in the Supplementary File.
