# Supplementary material for: Predictive value of D4Z4 methylation levels for phenotypic heterogeneity and disease progression in Facioscapulohumeral Muscular Dystrophy with borderline D4Z4 repeat units: a retrospective cohort study
Source: PeerJ. 2026 Apr 2;14:e21043. doi: 10.7717/peerj.21043 (PMC13050516; doi:10.7717/peerj.21043)
Supplement: Supplemental Table 1 [file peerj-14-21043-s001.docx]

**Supplementary Table 1. Comparison between hypomethylation and hypermethylation patients at baseline and follow-up based on CpG6 methylation levels.**

| **Subjects** | **Baseline** | | ***p* value** | **Follow-up** | | ***p* value** |
| --- | --- | --- | --- | --- | --- | --- |
|  | **Hypomethylation subgroup** | **Hypermethylation subgroup** |  | **Hypomethylation subgroup** | **Hypermethylation subgroup** |  |
| **Demographics** |  |  |  |  |  |  |
| Participants, n | 31 | 21 |  |  |  |  |
| Gender, male, n (%) | 19 (61.3) | 8 (38.1) | 0.10^a^ |  |  |  |
| Age at first examination, y, median (range) | 39 (15–63) | 42 (12–76) | 0.191*^b^* |  |  |  |
| Age at last follow-up, y, median (range) |  |  |  | 44 (19–70) | 46 (17–84) | 0.173*^b^* |
| Time of follow-up, y, median (range) |  |  |  | 5 (1–10) | 5 (3–9） | 0.516*^b^* |
| **Motor function** |  |  |  |  |  |  |
| FSHD clinical score (0–15) | 5 (0–12) | 1 (0–8) | **0.004***^b^* | 6 (0–14) | 1 (0–9) | **0.006***^b^* |
| CSS (0–5) | 3.0 (0–4.0) | 1.0 (0–3.5) | **0.002***^b^* | 3.0 (0–4.5) | 1.0 (1.0–4.0) | **0.003***^b^* |
| Age-corrected CSS ( 0–10000) | 129.63 (0–400) | 43.49 (0–233.33) | **0.001***^b^* | 120.0 (0–315.79) | 38.46 (0–189.19) | **0.001***^b^* |
| Lower extremity involvement, n (%) | 16 (51.6) | 6 (28.6) | 0.099^a^ | 18 (58.1) | 6 (28.6) | **0.036**^a^ |
| Independent ambulation loss, n (%) | 0 (0) | 0 (0) | - | 2 (6.5) | 0 (0) | 0.509^a^ |

^a^ *p* values were based on χ2 test (or Fisher’s exact test) for frequencies. ^b^ *p* value were based on Mann-Whitney *U* test for continuous variables. Values in bold indicate *p* value < 0.05.
